# Hospital admissions for treatment of ruptured and unruptured cerebral aneurysms within the Brazilian National Health System, 2009-2018: a descriptive study

**DOI:** 10.1590/S2237-96222022000200020

**Published:** 2022-10-03

**Authors:** Rossana Machado Sarmento, Roger dos Santos Rosa

**Affiliations:** 1 Universidade Federal do Rio Grande do Sul, Programa de Pós-Graduação em Saúde Coletiva, Porto Alegre, RS, Brazil; 2 Universidade Federal do Rio Grande do Sul, Departamento de Medicina Social, Porto Alegre, RS, Brazil

**Keywords:** Intracranial Aneurysm, Subarachnoid Hemorrhage, Epidemiology, Descriptive, Hospitalization, Health Expenditure, Brazilian National Health System

## Abstract

**Objective:**

To analyze hospital admissions for treatment of ruptured and unruptured cerebral aneurysms with embolization and brain microsurgery performed within the Brazilian National Health System (SUS), 2009-2018.

**Methods:**

This was a descriptive study, using data from the SUS’s Hospital Information System. Frequency of hospital admissions, procedures, use of intensive care unit (ICU), case fatality ratio and expenditures were described.

**Results:**

Of the 43,927 hospital admissions, 22,622 (51.5%) resulted in microsurgery. Embolization and cerebral microsurgery were more frequent among females. Length of hospital stay with embolization procedure was 7.7 days (±9.0), and with microsurgery, 16.2 (±14.2) days, frequency of ICU admission, 58.6% and 85.3%, and case fatality ratio, 5.9% and 10.9% respectively. Of the total expenditure, USD 240 million, 66.3% corresponded to hospitalizations with embolization procedure.

**Conclusion:**

Hospital admissions with embolization procedure for treatment of cerebral aneurysms within the SUS showed a shorter length of stay, less frequent use of ICU and lower case fatality ratio, but higher expenditure when compared to brain microsurgery.

**Study contributions**

**Main results**

For treatment of cerebral aneurysms, hospitalizations with embolization were less frequent and showed shorter length of stay and use of ICU - although it presented higher costs - and lower case fatality ratio, when compared to brain microsurgeries.

**Implications for services**

Shorter length of hospital stay and use of ICU in hospitalizations with embolization procedures may be good indicators of the arrangement and organization of services, regarding the turnover and use of hospital and ICU beds.

**Perspectives**

When the distribution of embolization and cerebral microsurgery procedures in the Brazilian public health system (SUS) is verified, it is worth reflecting on how the access to these services for the population has been occurring in the Brazilian territory.

## Introduction

Subarachnoid hemorrhage, characterized by the extravasation of blood into the subarachnoid or leptomeningeal space, due to cerebral aneurysm rupture (ruptured aneurysm),^1^ and unruptured cerebral aneurysms comprise the group of cerebrovascular diseases listed in the International Statistical Classification of Diseases and Related Health Problems 10^th^ Revision, ICD-10 (codes I60 to I69) that stand out among circulatory system diseases (chronic non-communicable diseases), either due to high incidence and mortality, or due to the significant increase in the occurrence of cases.^2^

A review of treatment options for intracranial aneurysm in the United States encompassed three studies conducted in 1998, 1999 and 2002, and showed that brain aneurysms are present in roughly 5% of the population, typically occur during adulthood, and rupture risk increases with age.^3^ Nontraumatic subarachnoid hemorrhage, secondary to cerebral aneurysm rupture, represents 5% to 15% of all strokes (cerebrovascular accident, CVA)^1^ and stands out in the group of cerebrovascular diseases due to the high mortality rate, ranging from 2 to 16 deaths per 100,000 inhabitants worldwide.^4^

Treatment for unruptured cerebral aneurysms, as well as subarachnoid hemorrhage (due to cerebral aneurysm rupture), is performed by means of surgical or endovascular techniques.^5^ Taking into consideration that the two main treatment options for this health problem within the Brazilian National Health System (*Sistema Único de Saúde* - SUS) are brain microsurgery and embolization, procedures that involve significant expenditure,^6^ the study aimed to analyze hospital admissions for the treatment of ruptured and unruptured cerebral aneurysms regarding the performance of embolization or brain microsurgery within the SUS, between 2009 and 2018.

## Methods

This was a retrospective, descriptive study, based on hospitalizations occurred within the SUS, in Brazil, between 2009 to 2018, registered on the SUS’s Hospital Information System (*Sistema de Informações Hospitalares do Sistema Único de Saúde* - SIH/SUS).

The study population was comprised of individuals whose main diagnosis was ruptured cerebral aneurysm (nontraumatic subarachnoid hemorrhage) or unruptured cerebral aneurysm, treated by means of embolization or cerebral microsurgery.

All hospital admission authorizations (*autorizações de internação hospitalar* - AIHs) with main diagnosis of subarachnoid hemorrhage (due to cerebral aneurysm rupture), according to ICD-10 I60 (including all its subdivisions: x.0 to x.9), or unruptured cerebral aneurysm, which is provided for in the ICD-10 I67.1 code, were included.

The types of treatments for the cases registered in the AIHs were analyzed based on the codes of procedures and treatment (embolization or brain microsurgery) included in the SUS Table of Procedures, Medicines, Orthotics, Prosthetics and Special Materials. The procedures analyzed in this study corresponded to Group 4, "Surgical Procedures", of this table. Within the subdivisions of Group 4, the "Subgroup 03 - Central and Peripheral Nervous System Surgery" was analyzed. After selecting this subgroup, the next filter enabled the choice of procedures to be observed. At this level, the table uses code 04 for the group of procedures that comprises the vascular neurosurgery treatment (microsurgery), and code 07 for the group of procedures that comprises the neuro-endovascular treatment (embolization).

Thus, 28 procedure codes were obtained: 12 related to microsurgery and 16 related to embolization. Among the 12 microsurgery codes, seven (040304003-5, 040304004-3, 040304007-8, 040304009-4, 040304010-8, 040304011-6 and 040304012-4) related to the main diagnoses analyzed, were selected. Of the 16 embolization codes, nine (040307002-3, 040307003-1, 040307004-0, 040307005-8, 040307006-6, 040307007-4, 040307014-7, 040307015-5 and 040307016-3) related to the main diagnoses analyzed, were selected.

The study variables were: sex (male; female); age (age groups between under 1 year and 80 years or over, organized every 5 years); length of stay (in days); use of intensive care unit (yes; no); occurrence of death (yes; no); and total hospitalization expenditure in Brazilian real (BRL).

Data on AIHs corresponded to compressed files (RD format) and were extracted on August 15, 2020. Data were checked and tabulated using a tabulator (TabNet), available at the Brazilian National Health System Information Technology Department (*Departamento de Informática do SUS* - DATASUS), of the Ministry of Health, via the TabWin application, which enables more advanced tabulations of files captured in RD format.^7^

Data analysis was performed using the Excel program®.

Average annual hospitalization rates were calculated, by sex and age group, for the period 2009-2018, dividing the number of hospitalizations occurred in the year by the projections of the resident population for each year, based on data from the 2010 population Census, multiplied by 1 million inhabitants.^8^

Absolute and relative frequencies of the type of procedure according to the main diagnosis (subarachnoid hemorrhage/ICD-10 I60; unruptured cerebral aneurysm/ICD-10 I67.1), stratified by sex and age group, were described.

The mean length of hospital stay, in days, by type of procedure, were obtained by calculating the ratio between the sum of the days that the cases studied spent in the hospital and the respective number of hospital admissions, according to sex and age group.

The relative frequency of ICU admission was calculated by dividing the number of ICU admissions, in days, by the total number of hospitalizations for each age group, multiplied by 100, and in-hospital case fatality ratio, by type of procedure, obtained by dividing the total number of hospital deaths for each procedure by the total number of procedures (embolization or brain microsurgery) multiplied by 100.

Hospitalization costs for each year were calculated in BRL and later converted to the average annual value of the daily exchange rate against USD. Initially, the daily sales values of the U.S. dollar were obtained from the website of the Brazilian Central Bank’s Time Series Management System, regarding the period from 2009; then, the average value of the USD for each year was calculated.^9^ Subsequently, the total annual values were converted by sex and age group. Thus, for each procedure group (embolization and brain microsurgery), the total and average expenditures were classified according to sex and age group.

The compressed files (RD) of SIH/SUS are in the public domain, made available by the Ministry of Health on the Internet in a format that ensures confidentiality and prevents the identification of subjects. Therefore, the study was exempted from approval of a Research Ethics Committee/National Research Ethics Committee (CEP/CONEP), as recommended by the National Health Council (*Conselho Nacional de Saúde* - CNS).

## Results

In Brazil, between 2009 and 2018, within the SUS, 43,927 hospitalizations for treatment of ruptured cerebral aneurysm (non-traumatic subarachnoid hemorrhage) and unruptured cerebral aneurysm, by means of embolization or cerebral microsurgery were registered. Embolization was performed in 21,305 hospitalizations (48.5%), and the hospitalization rate was 10.6 per 1 million inhabitants/year, while brain microsurgery was performed in 22,622 hospitalizations (51.5%), and the hospitalization rate was 11.3 per 1 million inhab./year (Table 1).

**Table 1:** Number of hospitalizations for subarachnoid hemorrhage (ruptured cerebral aneurysm) and unruptured cerebral aneurysm within the Brazilian National Health System and hospitalization rates (per million inhabitants/year), according to embolization procedure or brain microsurgery, by sex and age group, Brazil, 2009-2018

Age group (in years)	Embolization (hospitalization per million inhabitants/year)						Brain microsurgery (hospitalization per million inhabitants/year)					
	Male		Female		Total		Male		Female		Total	
	n	Rate	n	Rate	n	Rate	n	Rate	n	Rate	n	Rate
< 1	11	0.7	19	1.3	30	1.0	9	0.6	10	0.7	19	0.6
1-4	34	0.6	8	0.1	42	0.4	9	0.1	7	0.1	16	0.1
5-9	16	0.2	14	0.2	30	0.2	27	0.3	12	0.2	39	0.3
10-14	49	0.6	23	0.3	72	0.4	42	0.5	46	0.6	88	0.5
15-19	127	1.5	52	0.6	179	1.0	131	1.5	76	0.9	207	1.2
20-24	174	2.0	112	1.3	286	1.7	143	1.6	140	1.6	283	1.6
25-29	171	2.0	236	2.7	407	2.4	239	2.8	279	3.2	518	3.0
30-34	288	3.5	470	5.6	758	4.6	354	4.3	569	6.8	923	5.6
35-39	358	4.9	868	11.2	1,226	8.1	486	6.7	1,020	13.4	1,506	10.1
40-44	515	7.8	1,601	22.8	2,116	15.5	744	11.3	1,737	24.9	2,481	18.3
45-49	744	12.3	2,332	35.9	3,076	24.5	926	15.4	2,640	40.8	3,566	28.6
50-54	778	14.6	2,534	43.3	3,312	29.6	1,040	19.9	2,721	47.2	3,761	34.2
55-59	758	17.2	2,435	48.9	3,193	34.0	992	22.9	2,398	49.2	3,390	36.9
60-64	618	17.6	1,990	49.4	2,608	34.6	760	22.3	1,869	47.3	2,629	35.7
65-69	410	15.8	1,481	47.9	1,891	33.2	455	17.9	1,169	38.7	1,624	29.1
70-74	249	13.3	902	38.4	1,151	27.2	241	13.2	662	28.8	903	21.8
75-79	114	9.0	489	28.6	603	20.2	129	10.6	322	19.4	451	15.6
≥ 80	55	4.2	270	12.7	325	9.4	71	5.6	147	7.1	218	6.5
Total	5,469	5.6	15,836	15.5	21,305	10.6	6,798	7.0	15,824	15.5	22,622	11.3

Most hospitalizations occurred among female (72.0%), with 15,836 (74.3%) of these hospitalizations for embolization and 15,824 (69.9%) for microsurgery. Regardless of the type of procedure performed, the highest rates per million inhabitants/year were observed among people in the 40 to 44, 45 to 49, 50 to 54, 55 to 59, and 60 to 64 age groups. Rates have increased nearly twice, for both sexes and both causes of hospitalization (embolization and brain microsurgery), in the following age groups: 40 to 44 (15.5 and 18.3), 45 to 49 (24.5 and 28.6), 50 to 54 (29.6 and 34.2), 55 to 59 (34.0 and 36.9) and 60 to 64 years old (34.6 and 35.7), when compared to the age groups under 40 years old (Table 1).

Among individuals hospitalized due to cerebral aneurysms and undergoing embolization, the most frequent main diagnosis was subarachnoid hemorrhage (due to cerebral aneurysm rupture), which represented 61.0% of hospitalizations (n = 12,984), while unruptured cerebral aneurysms corresponded to 39.0% (n = 8,321) of that total (Table 2). As for hospitalizations due to brain microsurgery, 64.3% (14,535) of them had as main diagnosis subarachnoid hemorrhage (due to cerebral aneurysm rupture), while for 35.7% (8,087) of hospitalizations for this procedure, the main diagnosis was unruptured cerebral aneurysm (Table 2).

**Table 2:** Cause of hospitalization for subarachnoid hemorrhage (ruptured cerebral aneurysm) and unruptured cerebral aneurysm within the Brazilian National Health System, according to embolization procedure or brain microsurgery, by age group, Brazil, 2009-2018

Age group (in years)	Embolization				Brain microsurgery			
	Subarachnoid hemorrhage (ruptured cerebral aneurysm)		Unruptured cerebral aneurysm		Subarachnoid hemorrhage (ruptured cerebral aneurysm)		Unruptured cerebral aneurysm	
	n	%	n	%	n	%	n	%
< 1	9	0.1	21	0.3	14	0.1	5	0.1
1-4	21	0.2	21	0.3	10	0.1	6	0.1
5-9	16	0.1	14	0.2	30	0.2	9	0.1
10-14	49	0.4	23	0.3	56	0.4	32	0.4
15-19	106	0.8	73	0.9	147	1.0	60	0.7
20-24	198	1.5	88	1.1	189	1.3	94	1.2
25-29	266	2.0	141	1.7	357	2.5	161	2.0
30-34	495	3.9	263	3.2	623	4.3	300	3.7
35-39	783	6.0	443	5.3	996	6.9	510	6.3
40-44	1,337	10.3	779	9.4	1,595	11.0	886	11.0
45-49	1,926	14.8	1,150	13.7	2,271	15.6	1,295	16.0
50-54	1,991	15.3	1,321	15.8	2,391	16.4	1,370	16.9
55-59	1,881	14.5	1,312	15.7	2,137	14.7	1,253	15.5
60-64	1,558	12.0	1,050	12.5	1,632	11.2	997	12.3
65-69	1,079	8.3	812	9.8	1,026	7.1	598	7.4
70-74	689	5.3	462	5.6	584	4.0	319	3.9
75-79	361	2.8	242	2.9	312	2.1	139	1.7
≥ 80	219	1.7	106	1.3	165	1.1	53	0.7
Total	12,984	100.0	8,321	100.0	14,535	100.0	8,087	100.0

Overall average length of hospital stay for embolization was 7.7 days (±9.0), 7.9 days (±9.2) for males and 7.6 days (±8.9) for females, while for brain microsurgery, the average length of hospital stay was 16.2 days (±14.2), 15.9 days (± 14.2) for males and 16.3 days (±14.2) for females (Table 3).

**Table 3:** Mean length of hospital stay (in days) due to subarachnoid hemorrhage (ruptured cerebral aneurysm) and unruptured cerebral aneurysm within the Brazilian National Health System, according to embolization procedure or cerebral microsurgery, by gender and age group, Brazil, 2009-2018

Age group (in years)	Embolization mean (standard deviation)			Brain microsurgery mean (standard deviation)		
	Male	Female	Total	Male	Female	Total
< 1	6.2 (7.5)	13.4 (15.7)	10.7 (13.6)	9.8 (6.6)	17.3 (12.5)	13.7 (10.6)
1-4	9.1 (14.9)	3.8 (3.1)	8.1 (13.6)	5.7 (3.7)	6.3 (3.4)	5.9 (3.5)
5-9	6.5 (5.1)	7.6 (10.1)	7.0 (7.7)	11.3 (12.0)	8.1 (5.9)	10.3 (10.6)
10-14	6.9 (8.8)	6.3 (5.8)	6.7 (7.9)	15.6 (14.9)	15.2 (12.5)	15.4 (13.6)
15-19	7.2 (7.7)	8.4 (9.3)	7.5 (8.2)	14.3 (12.4)	14.3 (11.1)	14.3 (11.9)
20-24	7.9 (8.4)	8.9 (9.2)	8.3 (8.7)	12.4 (9.0)	16.8 (15.9)	14.6 (13.1)
25-29	8.0 (11.0)	7.7 (9.9)	7.8 (10.4)	15.2 (13.0)	14.9 (11.7)	15.1 (12.3)
30-34	7.5 (8.0)	7.8 (7.7)	7.7 (7.8)	14.2 (10.9)	15.7 (11.7)	15.1 (11.4)
35-39	8.7 (8.6)	7.3 (7.8)	7.7 (8.1)	14.8 (11.1)	14.8 (11.3)	14.8 (11.2)
40-44	8.6 (10.7)	7.3 (7.5)	7.6 (8.4)	15.6 (11.8)	16.3 (13.1)	16.1 (12.8)
45-49	7.6 (8.9)	7.4 (8.5)	7.5 (8.6)	16.0 (14.6)	15.8 (14.6)	15.8 (14.6)
50-54	8.1 (9.2)	7.3 (8.5)	7.5 (8.7)	16.0 (13.3)	16.4 (13.5)	16.3 (13.5)
55-59	8.0 (9.5)	7.2 (8.1)	7.4 (8.5)	16.5 (14.3)	16.4 (14.2)	16.4 (14.2)
60-64	8.4 (9.7)	7.9 (9.7)	8.0 (9.7)	18.1 (18.2)	17.0 (15.7)	17.3 (16.4)
65-69	7.0 (7.7)	7.6 (9.4)	7.4 (9.1)	17.2 (16.3)	17.6 (16.2)	17.5 (16.2)
70-74	6.9 (8.0)	8.0 (10.2)	7.8 (9.8)	16.2 (18.5)	17.7 (15.4)	17.3 (16.3)
75-79	8.5 (10.3)	9.0 (12.6)	8.9 (12.2)	14.9 (13.6)	17.8 (14.9)	17.0 (14.6)
≥ 80	6.7 (5.4)	8.9 (11.3)	8.6 (10.6)	10.0 (10.6)	17.0 (17.3)	14.7 (15.7)
Total	7.9 (9.2)	7.6 (8.9)	7.7 (9.0)	15.9 (14.2)	16.3 (14.2)	16.2 (14.2)

Of the hospitalizations due to embolization, 12,495 (58.6%) had a record of ICU admission and of these, 1,261 died (case fatality ratio was 5.9%). Among hospitalizations for brain microsurgery, 19,302 (85.3%) had a record of ICU admission and 2,455 died (case fatality ratio was 10.8%). It is worth emphasizing that the cause of death was similar, regardless of the treatment adopted. Among hospitalizations for subarachnoid hemorrhage due to ruptured cerebral aneurysm, there were 973 (77.2%) deaths among individuals undergoing embolization and 1,814 (73.9%) deaths among those hospitalized for brain microsurgery; and of hospitalizations for unruptured cerebral aneurysm, 288 (22.8%) of deaths occurred in individuals undergoing embolization, and 641 (26.1%) in those who underwent brain microsurgery (Table 4).

**Table 4:** Use of intensive care unit (ICU) and hospital deaths due to subarachnoid hemorrhage (ruptured cerebral aneurysm) and unruptured cerebral aneurysm within the Brazilian National Health System, according to embolization procedure or brain microsurgery, by age group, Brazil, 2009-2018

Age group (in years)	Embolization						Brain microsurgery					
	Use of ICU		Hospital deaths				Use of ICU		Hospital deaths			
	Yes (%)	No (%)	Subarachnoid hemorrhage (ruptured aneurysm)		Unruptured cerebral aneurysm		Yes (%)	No (%)	Subarachnoid hemorrhage (ruptured aneurysm)		Unruptured cerebral aneurysm	
			n	%	n	%	n	%	n	%	n	%
< 1	17 (56.7)	13 (43.3)	-	0.0	1	100.0	9 (47.4)	10 (52.6)	-	0.0	-	0.0
1-4	29 (69.0)	13 (31.0)	-	0.0	-	0.0	13 (81.3)	3 (18.8)	-	0.0	-	0.0
5-9	18 (60.0)	12 (40.0)	-	0.0	-	0.0	28 (71.8)	11 (28.2)	3	60.0	2	40.0
10-14	42 (58.3)	30 (41.7)	3	100.0	-	0.0	73 (83.0)	15 (17.0)	3	60.0	2	40.0
15-19	93 (52.0)	86 (48.0)	2	50.0	2	50.0	173 (83.6)	34 (16.4)	12	92.3	1	7.7
20-24	167 (58.4)	119 (41.6)	6	85.7	1	14.3	216 (76.3)	67 (23.7)	20	71.4	8	28.6
25-29	246 (60.4)	161 (39.6)	6	46.2	7	53.8	435 (84.0)	83 (16.0)	29	82.9	6	17.1
30-34	456 (60.2)	302 (39.8)	18	78.3	5	21.7	795 (86.1)	128 (13.9)	53	85.5	9	14.5
35-39	718 (58.6)	508 (41.4)	42	75.0	14	25.0	1,275 (84.7)	231 (15.3)	97	78.2	27	21.8
40-44	1,226 (57.9)	890 (42.1)	79	79.0	21	21.0	2,092 (84.3)	389 (15.7)	140	76.1	44	23.9
45-49	1,772 (57.6)	1,304 (42.4)	96	80.0	24	20.0	3,060 (85.8)	506 (14.2)	242	75.6	78	24.4
50-54	1,979 (59.8)	1,333 (40.2)	135	81.8	30	18.2	3,213 (85.4)	548 (14.6)	273	76.7	83	23.3
55-59	1,869 (58.5)	1,324 (41.5)	149	77.6	43	22.4	2,923 (86.2)	467 (13.8)	275	67.7	131	32.3
60-64	1,524 (58.4)	1,084 (41.6)	141	77.5	41	22.5	2,255 (85.8)	374 (14.2)	221	68.4	102	31.6
65-69	1,117 (59.1)	774 (40.9)	108	74.5	37	25.5	1,398 (86.1)	226 (13.9)	191	77.3	56	22.7
70-74	677 (58.8)	474 (41.2)	72	72.0	28	28.0	782 (86.6)	121 (13.4)	120	72.3	46	27.7
75-79	351 (58.2)	252 (41.8)	59	74.7	20	25.3	383 (84.9)	68 (15.1)	80	76.2	25	23.8
≥ 80	194 (59.7)	131 (40.3)	57	80.3	14	19.7	179 (82.1)	39 (17.9)	55	73.3	20	26.7
Total	12,495 (58.6)	8,810 (41.4)	973	77.2	288	22.8	19,302 (85.3)	3,320 (14.7)	1,814	73.9	641	26.1

The total expenditure on hospitalizations and procedures in the period was USD 240,351,623.69, of which USD 159,336,383.05 (66.3%) on hospitalizations with embolization (average expenditure per hospitalization of USD 976.59) and USD 81,015,240.64 (33.7%) on hospitalization with brain microsurgery (average expenditure per hospitalization of USD 220.94) (Table 5).

**Table 5:** Total and average expenditures on hospitalizations for subarachnoid hemorrhage (ruptured aneurysm) and unruptured cerebral aneurysm within the Brazilian National Health System, according to embolization procedure or brain microsurgery, by sex and age group, Brazil, 2009-2018

Age group (in years)	Embolization (costs in US dollar - USD)				Brain microsurgery (costs in US dollar - USD)			
	Male	Female	Total	Average expenditure on hospitalization	Male	Female	Total	Average expenditure on hospitalization
< 1	60,662.13	86,738.66	147,400.78	457.77	23,366.39	29,125.32	52,491.70	201.12
1-4	272,019.42	43,766.11	315,785.53	931.52	18,357.37	19,244.33	37,601.70	395.81
5-9	115,524.11	83,129.95	198,654.06	941.49	90,646.64	29,414.79	120,061.43	297.92
10-14	336,152.78	102,219.83	438,372.61	909.49	137,137.61	156,155.51	293,293.12	216.61
15-19	909,550.01	310,596.08	1,220,146.08	903.81	410,970.62	251,453.85	662,424.47	223.72
20-24	1,203,336.27	733,888.95	1,937,225.23	818.78	513,331.42	458,604.15	971,935.57	235.56
25-29	1,122,342.55	1,542,995.43	2,665,337.99	834.74	773,992.39	920,918.55	1,694,910.94	217.41
30-34	2,142,698.01	3,625,589.61	5,768,287.62	990.94	1,189,486.35	1,952,331.08	3,141,817.43	225.37
35-39	2,694,410.36	6,524,494.98	9,218,905.34	977.30	1,661,164.30	3,474,294.26	5,135,458.56	229.65
40-44	3,904,366.70	12,655,126.69	16,559,493.39	1,028.80	2,639,064.69	5,986,917.96	8,625,982.65	216.27
45-49	5,723,536.53	18,285,697.49	24,009,234.02	1,044.42	3,222,435.57	9,375,679.86	12,598,115.42	223.42
50-54	5,909,483.16	18,981,337.90	24,890,821.06	999.87	3,666,396.03	9,802,157.27	13,468,553.30	220.26
55-59	5,625,969.27	17,886,358.89	23,512,328.16	997.47	3,650,969.87	8,747,180.65	12,398,150.52	222.94
60-64	4,559,882.96	14,521,390.53	19,081,273.48	911.02	2,777,313.87	6,754,502.66	9,531,816.54	209.61
65-69	3,079,936.81	11,151,412.00	14,231,348.81	1,011.61	1,696,979.17	4,431,675.77	6,128,654.94	215.75
70-74	1,793,283.84	6,588,605.90	8,381,889.74	938.20	881,815.23	2,645,945.02	3,527,760.25	226.34
75-79	843,960.64	3,57,557.43	4,417,518.07	823.70	459,920.40	1,350,578.90	1,810,499.30	235.99
≥ 80	417,354.22	1,925,006.84	2,342,361.07	842.88	244,043.68	571,669.12	815,712.80	254.43
Total	40,714,469.77	118,621,913.27	159,336,383.05	976.59	24,057,391.60	56,957,849.04	81,015,240.64	220.94

It could be seen higher costs on hospitalizations of females, compared to costs on hospitalizations of males, regarding both procedures. The values registered for hospitalization of females, with embolization, were USD 118,621,913.27 (74.4%), and with brain microsurgery, USD 56,957,849.04 (70.3%) (Table 5).

## Discussion

This study has found that, within the SUS, from 2009 to 2018, brain microsurgery, for the treatment of ruptured and unruptured cerebral aneurysms, was more frequent than embolization and, consequently, had the highest hospitalization rate per million inhabitants, per year. Hospitalization rates for ruptured and unruptured cerebral aneurysms were concentrated in the adult population. There was a higher number of hospital admissions, for both procedures, among females, and a prevalence of hospital admission whose main diagnosis was subarachnoid hemorrhage (ruptured cerebral aneurysm), regardless of the type of procedure adopted. The average length of stay in hospital and in the ICU, as well as in-hospital case fatality ratio, were higher in hospitalizations with brain microsurgery when compared to hospitalizations with embolization; the latter, however, had higher total and average expenditures.

Analysis of annual rates of hospitalization for cerebral aneurysm, according to the type of procedure within the SUS, showed slightly higher values for brain microsurgery, when compared to embolization. This result is different from that observed in a cohort study conducted in the United Kingdom, 18 years after the introduction of the embolization technique for treatment of nontraumatic subarachnoid hemorrhage in that country, where it was found that 85% of the cases of subarachnoid hemorrhage due to cerebral aneurysm rupture had undergone embolization.^10^

In agreement with other studies, the highest percentage of hospitalization for stroke was recorded in the population between 40 and 79 years of age, regardless of the type of procedure adopted, with emphasis on the highest occurrence of hospitalizations among the female population.^11^ ^-^ ^15^ Being female, being over 40 years of age, smoking status, using alcohol, using drugs and having chronic diseases (factors that have not been analyzed in this study) comprise a set of risk factors defining the profile of the population affected by subarachnoid hemorrhage due to ruptured and unruptured cerebral aneurysms.^16^ ^,^ ^17^

The literature, supported by several studies, suggests the hypothesis that the decline in hormone levels after menopause causes hemodynamic stress and vascular remodeling in women, favoring cerebral aneurysm formation, which, in turn, would explain the prevalence of female representativeness in these studies.^11^ ^,^ ^12^ Other authors add to this explanation the fact that women have an increased risk for vasospasm.^13^ On-screen analysis showed the prevalence of hospitalizations for subarachnoid hemorrhage due to cerebral aneurysm rupture, recognized as an acute neurological emergency, as it results from the potential of cerebral aneurysm rupture present in the population in a silent way.^3^ ^,^ ^18^

Regarding the overall average time for the treatment of ruptured and unruptured cerebral aneurysms, this study differs from other studies. A descriptive study conducted at the University Hospital of Madrid in 2017, when consulting medical records of individuals admitted between 1995 and 2015 and comparing the two treatment techniques, embolization and brain microsurgery, found an average length of hospital stay of 28 days for embolization and 32 days for brain microsurgery.

However, studies that did not distinguish between the treatment techniques adopted, such as an evaluative study of people after discharge from hospitalization for treatment of subarachnoid hemorrhage in 14 centers in the United Kingdom, in the period between 2011 and 2015,^19^ identified an overall median hospital stay of 15 days. Another study, conducted at the Columbia University Medical Center, with individuals admitted between 1996 and 2009, undergoing treatment for subarachnoid hemorrhage, found a median hospital length of stay of 14 days for survivors and 5.5 days among non-survivors.^20^

ICU admission between hospitalizations for treatment of ruptured and unruptured cerebral aneurysms was recorded in more than 50% of embolization procedures and brain microsurgeries. In addition, hospitalizations for ruptured cerebral aneurysms accounted for more than 70% of deaths recorded in embolization and brain microsurgery procedures. Although its frequency in the population is lower when compared to other brain hemorrhages, the severity of subarachnoid hemorrhage due to cerebral aneurysm rupture is recognized because it is one of the main causes of mortality. Mortality from ruptured cerebral aneurysms can reach 32% on the first day and 46% in the first week since the first symptom was reported.^1^ ^,^ ^5^

This study identified a higher in-hospital case fatality ratio among hospitalizations for brain microsurgery, when compared to those for embolization, differing from the findings of a study that identified an overall death rate of 18%: 14% of the total was related to embolization procedure, while 7% was related to brain microsurgery.^20^

Overall expenditure on embolization procedures was significantly higher than that of brain microsurgery among hospitalizations occurred within the SUS and recorded on the SIH/SUS. It is worth highlighting that during the study period, ministerial ordinances that changed the SUS Table of Procedures, Medicines and Orthotics, Prosthetics and Special Materials (*Tabela de Procedimentos, Medicamentos e Órtese, Prótese e Materiais Especiais do SUS* - SIGTAP) were published, and affected, specially, the values and quantities of materials used in the procedures.^21^ ^,^ ^22^ With regard to embolization, the value of the platinum spiral (material used in these procedures) established in the SUS Table of Procedures, Medicines, Orthotics, Prosthetics and Special Materials, was BRL 2,230.00 until April 2011, being reduced to BRL 1,350.00 on April 27, that same year.^21^ ^,^ ^22^ Regarding microsurgery, as of December 16, 2010, titanium alloy clips (material used in this type of procedure) have been included in this table, and they started to cost BRL 800.00 for the SUS; until then, the clips that comprised the table were made of cobalt-chromium alloy and its value was set at BRL 785.00.^21^ ^,^ ^22^

Despite these changes in the table values, it could be seen that the average cost per embolization procedure was higher than the average cost per brain microsurgery procedure. This is evident when the findings of this research are compared to those of a study that showed an average expenditure for the SUS, per embolization procedure, of BRL 17,261.00 in the period from 2010 to 2015, while the average expenditure on brain microsurgery for the system was BRL 8,078.80.^23^

It is noteworthy that the procedures analyzed in this study, and the expenses related to them, are provided for in the Ordinance of the Ministry of Health No. 1,161/GM, of July 7, 2005, when the National Health Policy for People with Neurological Disorders was established. In this context, among a set of actions that this policy encompasses, access to neurological, neuro-interventional and neurosurgery procedures is ensured as a way of achieving a positive impact on survival, morbidity and quality of life of individuals.^24^

Nevertheless, the results of this study show an inversely proportional relationship between cost and length of hospital stay when comparing embolization and brain microsurgery procedures: embolization recorded a higher expenditure, but a shorter length of hospital stay. A shorter length of stay for hospitalizations with embolization may be attributed to the fact that this procedure is less invasive.^4^ ^,^ ^13^ However, although brain microsurgery has presented a longer mean period in days of hospitalizations, it represented half of the average expenditure when compared to embolization. One of the possible justifications for this result lies in the unit value of the products used in each procedure. In addition, the amount of use of these materials, whose payment authorization is made by the SUS, also affects these values, with up to ten platinum spirals for each embolization procedure, while for brain microsurgery, three permanent clips and three temporary clips are provided. Thus, it is suggested that the expenditures identified in this research be used as a source for health economic evaluation studies.

Among the limitations of this study, we highlight those related to the administrative data source used (public files from hospital admission authorizations) (*autorizações de internação hospitalar* - AIHs), without a variable indicating the severity of the case at the time of hospital admission. Based on the database used, therefore, it was not possible to make inferences about the differences in mortality between the procedures observed, given that the AIHs do not present clinical information regarding hospital admission of individuals. Moreover, it was not possible to identify the cases that, after undergoing embolization, had presented complications and underwent microsurgery.

Taking these results, it can be concluded that embolization showed lower frequency, shorter length of hospital stay, lower percentage of use of ICU, lower hospital case fatality ratio and, however, higher total and average expenditure per hospitalization, when compared to microsurgery, for treatment of ruptured and unruptured cerebral aneurysms within the SUS.
